# Case Report: Integration between eye movement desensitization and reprocessing and cognitive therapy for autism spectrum disorder. Novel intervention protocol based on case formulation and brief review of literature

**DOI:** 10.3389/fpsyg.2026.1812438

**Published:** 2026-05-01

**Authors:** Maria Marino, Mariangela Pezone, Maria Pia Riccio, Raffaele Garotti, Annamaria Scapicchio, Isabel Fernandez, Carmela Bravaccio

**Affiliations:** 1 EMDR Italian Association, Varedo, Italy; 2 School of Cognitive Psychotherapy (SPC), Naples, Italy; 3 Residency Program of Child and Adolescent Neuropsichiatry, Department of Medical and Translational Sciences, Federico II University, Naples, Italy; 4 Federico II University Hospital, Maternal-Infant Department, Child and Adolescent Neuropsychiatry, Naples, Italy; 5 Department of Mental and Physical Health and Preventive Medicine, University of Campania "Luigi Vanvitelli", Naples, Italy; 6 Center for Research and Studies in Psychotraumatology, Milan, Italy; 7 Department of Medical and Translational Sciences, Child and Adolescent Neuropsychiatry, Federico II University, Naples, Italy

**Keywords:** autism spectrum disorder, EMDR, post-traumatic stress disorder, psychopathological vulnerability, relational trauma

## Abstract

Autism Spectrum Disorder (ASD) is a neurodevelopmental disorder characterized by persistent deficits in social communication and interaction, alongside restricted, repetitive, and stereotyped patterns of behavior and interests. Individuals on the autism spectrum exhibit increased psychopathological vulnerability, with a high incidence of comorbid conditions, including Post-Traumatic Stress Disorder and trauma-related symptoms. Trauma-related symptoms appear to be highly prevalent in ASD, though they are frequently underestimated. Furthermore, autistic individuals face numerous risk factors that increase their exposure to traumatic experiences and relational trauma. It is also likely that there is an autism-specific vulnerability regarding the perceived traumatic impact of certain events. This paper presents the clinical case of a young autistic woman with no intellectual or language impairment, comorbid social-performance anxiety, and pathogenic schemas associated with relational trauma. The patient was treated with Eye Movement Desensitization and Reprocessing (EMDR) therapy sessions, employing protocol modifications tailored to the ASD condition. Currently, studies on the use of EMDR in ASD are limited; the primary barrier to its application often stems from challenges in adapting the technique to the specific needs of autistic patients. Therefore, further research is essential—not only to evaluate the efficacy of EMDR in ASD but, crucially, to refine and adapt the standard protocol to address the specific requirements and challenges of this condition.

## 1 Introduction

Autism Spectrum Disorder (ASD) is a neurodevelopmental disorder characterized by socio-communicative and relational difficulties associated with restricted/repetitive patterns of behavior (American Psychiatric Association, 2022). The core symptoms are strongly correlated with and mediated by differences in information processing, executive functions, emotional and sensory regulation, and expose autistic people to increased psychopathological vulnerability (Marino, 2024). Different levels of severity characterise ASD and it is possible to stratify the disorder based on intellectual and language impairment. Severity level 1 refers to highest functioning individuals, without intellectual or language impairment. In these cases, during adolescence and adulthood, the “core” manifestations can become extremely nuanced, while differences in information processing and regulation remain more evident, with a predisposition to cognitive, emotional and sensory overload; consequently a greater vulnerability and incidence of regulation difficulties can occur (Marino, 2024; Fung, 2021; Attwood, 2007). Autistic people deal with a higher risk of adverse situations and possess a reduced coping capacity in stressful situations, so that stress vulnerability and lack of coping strategies should be considered an autism-correlated specificity (DePrince, 2005; Mazefsky et al., 2013; Rumball et al., 2020). Regarding increased vulnerability, Rumball et al. agree on extending the definition of trauma beyond the scope of the Diagnostic and Statistical Manual of Mental Disorders, Fifth Edition (DSM-5) for autistic people, incorporating events that are currently not described within the diagnostic criteria for Post-Traumatic Stress Disorder (PTSD) (Rumball et al., 2020). With respect to autism-related characteristics, the specificity is related also to differences in the nervous system, including autonomic functions, with greater and more stable activation of the sympathetic and parasympathetic (dorsovagal) branches of the autonomic nervous system (ANS) and a lesser integration and effectiveness of the ventrovagal response. This contributes to greater reactivity in autistic people in the autonomous detection of threat signals, and greater activation and persistence of the defense system (sympathetic and/or parasympathetic dorsovagal activation). This neurophysiological substrate may explain, in part, the greater presence and chronicity of states of hyperarousal (anxiety, flight reaction, angry behaviors, meltdown) or hypoarousal (fogginess, dissociation, freezing, depression, shutdown) and the related emotional and cognitive characteristics. Autistic people often remain in a state of chronic tension and anxiety and have little access to physiological sensations of safety and connection, with an impact on their response and coping with stress and on skills related to social connection (Inderbitzen, 2025). This increased vulnerability raises the incidence of chronic stress, anxiety and depressive symptoms, and can negatively influence the capacity to manage any future stressors, increasing the risk of re-victimization or re-traumatization (Marino, 2024; van Lobregt- Buuren et al., 2019). The differences in the regulation of the threat and security signal detection system and the high prevalence of trauma-related symptoms in ASD (Rumball et al., 2020; van Lobregt- Buuren et al., 2019; Fisher et al., 2022) suggest the utility of applying focused and specific intervention protocols for self-regulation and stabilization of a greater sense of security on a conscious and neuroceptive level and for trauma and related symptomatology. The EMDR (Eye Movement Desensitization and Reprocessing) is an 8-phase, bottom-up therapy based on the Adaptive Information Processing Model. It uses bilateral stimulation to reactivate the process of elaborating information that are dysfunctionally stored at a neurophysiological level following traumatic events (Shapiro, 1989). The work is structured in steps, beginning with the recognition of the most significant episodes in the patient’s expressed symptomatology and their identification as targets correlated with specific cognitive, emotional, and sensory elements. These elements are then worked on using bilateral stimulation until their processing and resolution, with reduction of perceived distress and associated negative cognitions (Shapiro, 2018). Originally used as a treatment for individuals with PTSD, EMDR has evolved into an integrated approach, demonstrating its efficacy not only for PTSD but also for a variety of psychopathological disorders and conditions related to relational trauma. To date, despite the increased vulnerability to trauma, data regarding the use of EMDR in psychotherapy for ASD are scarce, the integration of EMDR into psychotherapy for autism is still rarely utilized, and operative and generalizable guidelines within shared protocols have not yet been developed. Based on literature data and from our point of view, polyvagal theory and its application to working with ASD (Inderbitzen, 2025) offers useful strategies for increasing, self-regulating, and stabilizing a sense of security, even in relation to the characteristic emotional and sensory differences of autism. These strategies allow the patient to remain within their window of emotional tolerance (Siegel, 2021), maintaining the stability necessary to undertake and sustain psychotherapeutic work with the EMDR, helping to adapt this protocol to the specific neurological differences expressed in autism. Therefore, the case of a young autistic girl with no intellectual or language impairment is presented, for whom the integration of EMDR treatment and regulatory strategies based on the polyvagal theory into the cognitive therapeutic process proved useful for certain detected clinical comorbidities. Polyvagal theory tools have been used as heuristics to support patient self-regulation, in the preparation and stabilization phase, in therapeutic work, and during the administration of EMDR, within a broader vision of autism as a condition related to differences in information processing. An EMDR protocol adapted for autistic people is proposed.

## 2 Case presentation

We present the case of a 23-year-old female patient with a confirmed diagnosis of ASD according to DSM-5 criteria. The patient started psychotherapeutic intervention during adolescence, following the diagnosis of ASD, associated with Specific Learning Disorder, deficits in executive control and pronounced socio-performance anxiety. The diagnosis was made according to the standards of the guidelines and was conducted through a specific psychometric assessment, including also the *Autism Diagnostic Observation Schedule* and the *Autism Diagnostic Interview-Revised.* The patient’s clinical history is unremarkable for significant medical comorbidities (epilepsy and/or genetic syndromes). At the time of the assessment, the Intelligence Quotient, evaluated using Raven’s Matrices, was found to be within the normal range (IQ = 96). In the initial phase of treatment, beyond establishing a therapeutic alliance, the focus was placed on the identification of pathogenic pattern and core beliefs, alongside the patient’s long-term life objectives. These objectives centered on achieving a sense of acceptance and inclusion, fostering spontaneous expression, increasing autonomy, and developing self-efficacy in both personal and social domains. Subsequently, the therapeutic work incorporated sessions dedicated to support awareness, discussing the patient’s neurodivergence and developing specific management strategies, given their significance as independent variables in the internal manifestation of the disorder (Marino, 2024). In accordance with the patient’s needs and in accordance with the biopsychosocial model of neurodiversity, we agreed to use the term autistic person to refer to the patient’s neurodivergent condition (Vivanti, 2020).

**Figure 1:**
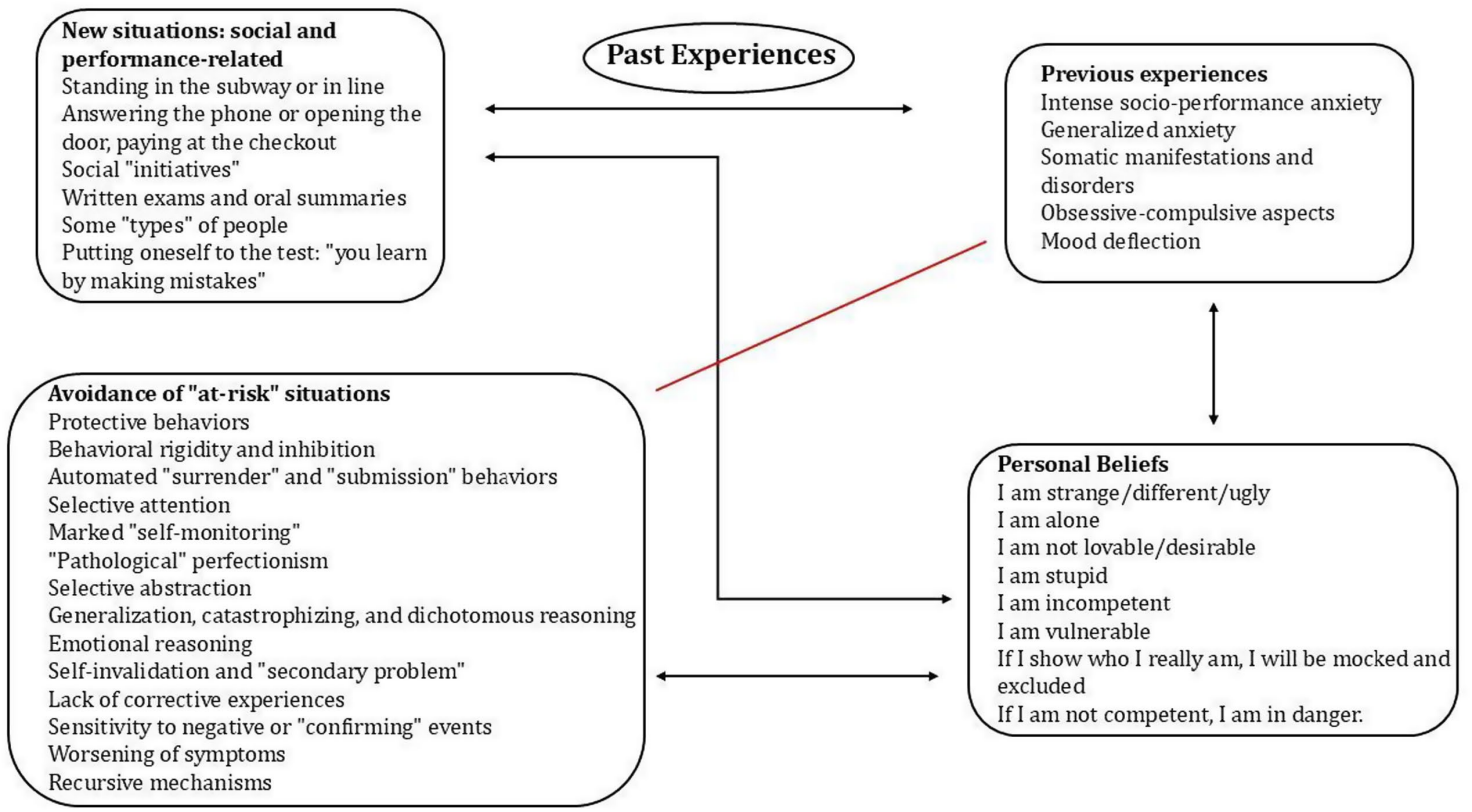
Elements of the internal profile of the patient’s psychological functioning and their dynamic correlation.

**Figure 2:**
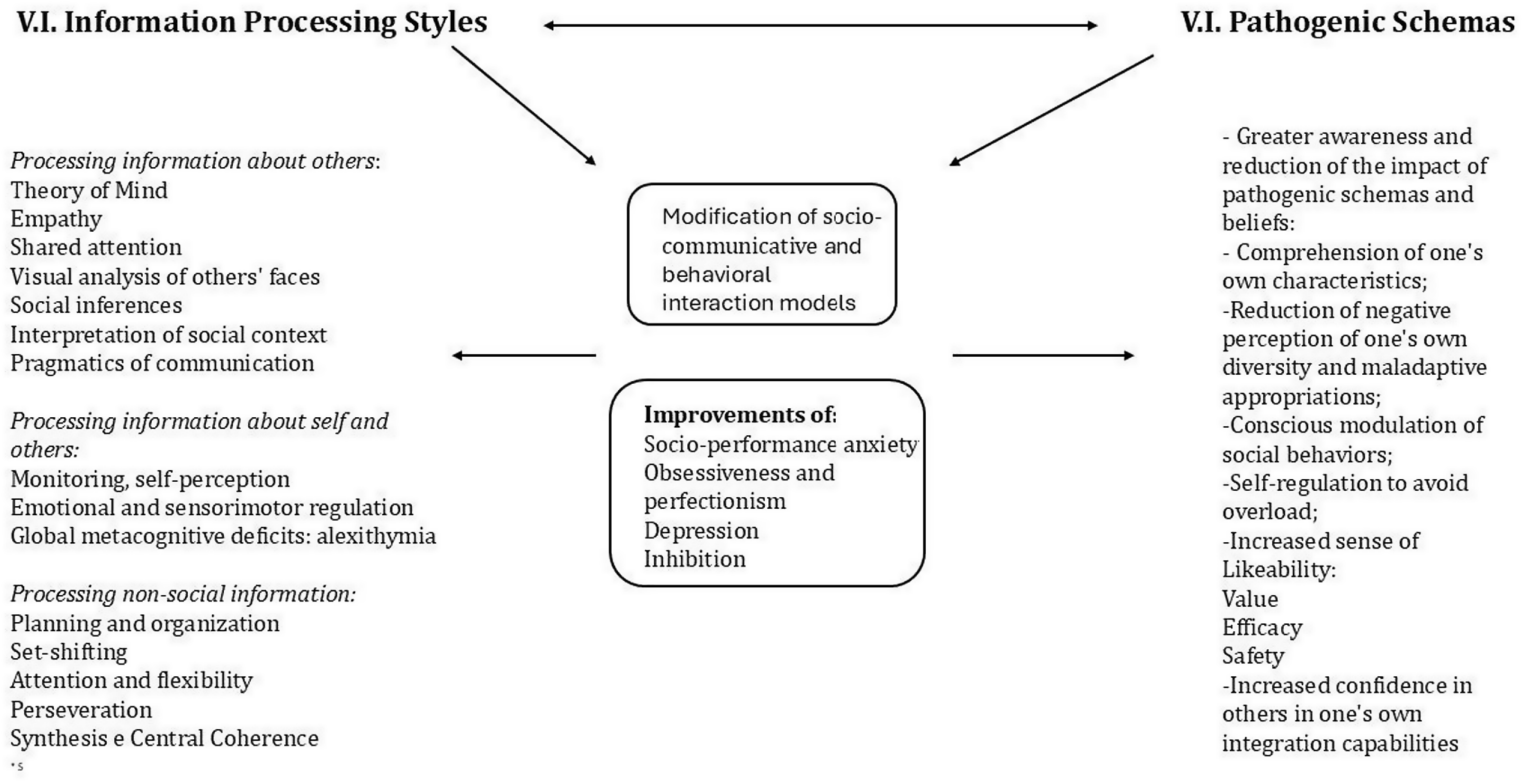
Scheme representing the psychoeducational and psychotherapeutic work of integrating pathogenic patterns and variables related to neurodivergent processes and characteristics.

Figure 1 summarizes the components of the patient’s profile. The emotional and behavioral reaction, consistent with a negative and threatening appraisal of social and performance situations, was mediated by numerous dysfunctional cognitive biases and processes. These were underpinned by negative schemas and beliefs pertaining to the domains of self-defectiveness, unlovability, safety, control over choices, and autonomy. The influence of recursive vicious-cycle mechanisms and severe self-criticism, coupled with a perceived helplessness in modifying her situation, contributed to the generation of somatic symptoms and depressed mood. Psychotherapy focused on reducing symptoms of anxiety and depression and reducing avoidance behaviors. Identifying biases, pathogenic patterns, and critical reasoning increased cognitive remodeling. Gradual exposure with response prevention modified behavior, promoting exploration. At the same time the psychotherapeutic intervention gradually integrated work on the identification, awareness, and management of the patient’s neurodivergent characteristics, particularly those related to metacognitive, personal, interoceptive, sensory, and executive processes, all of which influence emotional regulation and social/interpersonal behaviors. Figure 2 summarizes the integration work between pathogenic schemas and variables related to neurodivergent processes and characteristics. EMDR was integrated into the therapeutic process following the patient’s clinical worsening characterized by acute anxiety and behavioral dysregulation. This deterioration was related to a work experience that required her to stay abroad.

This experience triggered a pronounced anxious reaction characterized by the implementation of dysfunctional control behaviors, difficulties in managing unforeseen events, and maladaptive coping strategies. On the way back, during the therapeutic intervention, the patient demonstrated a good capacity for critical and constructive self-reflection regarding her reactions and an ability to identify the contingent factors and their impact (the overload of stimuli, together with the sense of insecurity in coping with new experiences, has worn the patient down, contributing to the sense of overwhelming and the subsequent meltdown). Simultaneously, she reported feeling overwhelmed by emotions so intense that she was unable to utilize the strategies previously established in therapy. These aspects suggested the presence of underlying traumatic elements influencing the patient’s response, thereby indicating the utility of incorporating more experiential strategies better suited to the expressed reaction. Consequently, a shift was made to a predominantly bottom-up approach utilizing the EMDR protocol.

## 3 Details on the therapeutic intervention

**Figure 3:**
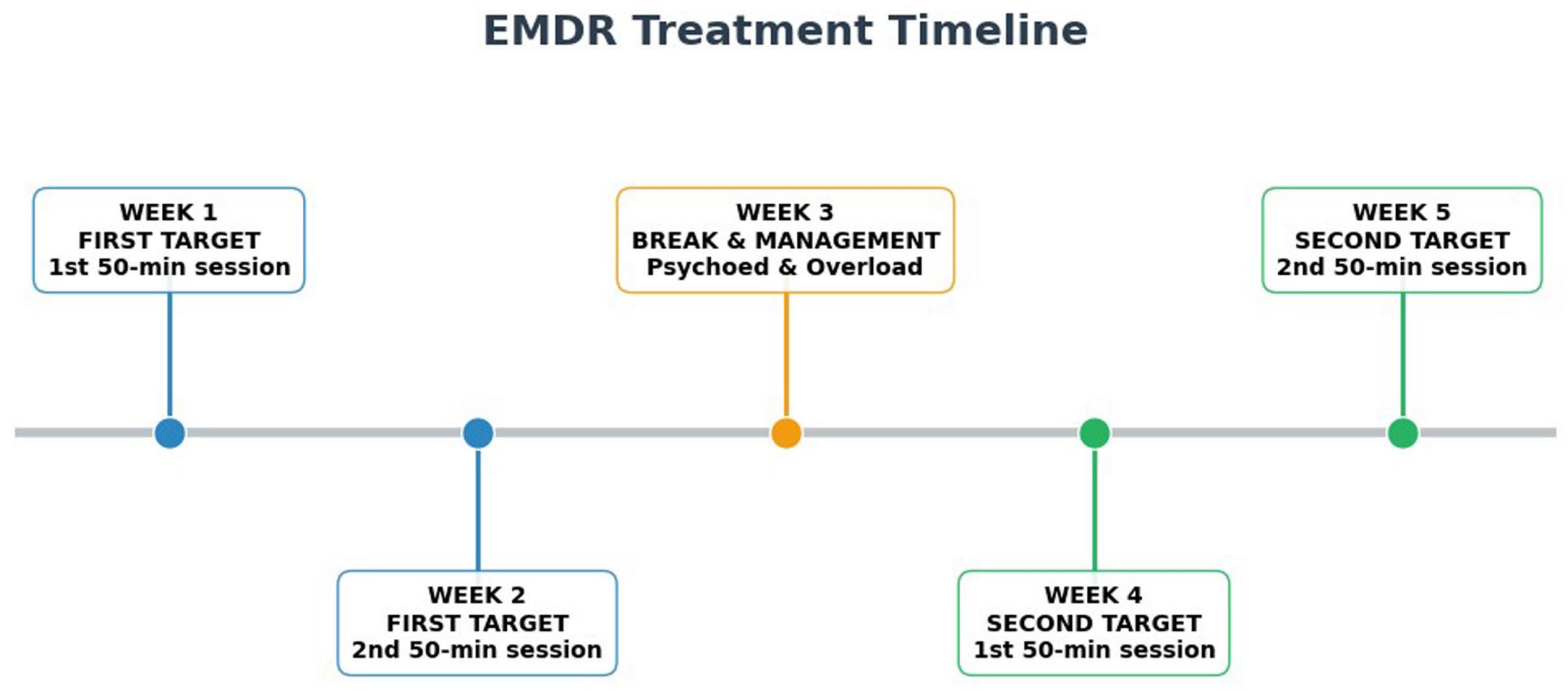
EMDR sessions timeline.

The work protocol was clearly explained to the patient in all its phases. The EMDR protocol was adapted to the patient’s neurodivergent characteristics and integrated with strategic application derived from the Polyvagal Theory (Porges, 2011). The patient exhibited high levels of tension and hyperarousal, which persisted over time, even in safe conditions. This characteristic of hyperarousal, whether sympathetic or parasympathetic, is described in autistic people and correlates with hyperactivation of the threat system, an altered perception of safety, a tendency toward chronic anxiety, and increased occurrence of acute agitation or freezing (Inderbitzen, 2025). This has a significant impact on the life experiences of neurodivergent people and makes trauma work more complex, amplifying its effects. Working on the perception of safety was therefore necessary. Identifying sensory activation markers, breathing exercises, body awareness exercises, accessing resources through imagery, mindfulness exercises, and the use of imagery were used to help the patient restore a greater sense of safety, bringing her back within her window of tolerance (Siegel, 2021). The patient was subsequently able to self-regulate using these strategies, reaching the level of stabilization necessary for the use of EMDR. Understanding the patient’s psychological profile, we introduced EMDR work strategically and effectively to achieve the most important target. We worked for five weeks, in four EMDR sessions, each lasting 50 min. Each target required two sessions. Between the two targets, a one-week interval was planned and agreed upon for a psychoeducational review, useful for synthesis and reprocessing, and to allow the patient to recover, given the emotional, cognitive, and sensory overloading experienced. A timeline figure is included (Figure 3) The EMDR protocol was then, in turn, adapted to the specific characteristics of the patient, especially in relation to the metacognitive and sensorial characteristics and, always, in relation to the maintenance of an optimal activation state, within the patient’s tolerance window (connected state, ventrovagal engagement). The floatback technique was used to identify past relational events with a traumatic valence, asking the patient to focus on the identified image and associated sensations. Based on clinical reasoning, floatback was used in a targeted manner, to avoid overloading the patient, simplifying and adapting the assessment. The patient identified two targets, dating back to family episodes experienced during her childhood, on which working to highlight the correlated characteristics in terms of bodily sensations, emotions, and negative cognitions:

1. Target Event: “I am about 5 years old, I am in the car with my mother, going to a place unknown to both of us…”

- Worst Image: “Us getting lost and Mom’s nervous and scared face asking me what we should do, and me small in the seat”;
- Negative Cognition: “*I am in danger, I am incapable, I have no control, and I cannot trust”* (Cognition domain: self-defectiveness; safety; control);
- Positive Cognition: I am safe; Validity of Cognition (VoC): 3;
- Emotions: “*I am afraid, I am in panic and confusion”*; Subjective Units of Disturbance (SUD): 8;
- Body Location: “My heart and chest are bursting”

1. Target Event: “I am about 5-6 years old; when I call Mom, she suddenly becomes irritable for no reason and scolds me. Dad, to make her ‘calm down,’ tells me to apologize to her…”

- Worst Image: “Me going to Dad trustingly and him not defending me but telling me ‘apologize, it will pass’…”
- Negative Cognition: “I cannot trust anyone, and I cannot express myself” (Domains: control; safety);
- Positive Cognition: I can choose who to trust, and I can express myself freely; VoC: 3;
- Emotions: “I am afraid, I feel sad, lonely, I have fear and apprehension… I am also angry…” SUD: 7;
- Body Location: “Heat and heaviness in my chest and the urge to cry”

During the target definition, it was decided to be tolerant regarding the coexistence of multiple negative cognitions, without insisting on the patient isolating only one, as per the classic protocol. This decision was based on clinical reasoning and motivated by the need, given the patient’s characteristics, to maintain a good balance between activation, emotional and cognitive load, and the fluidity of information processing necessary for effective work with EMDR, thereby avoiding excessive stress for the patient. In defining the target and in the subsequent elaboration, the clinician paid attention to the cognitive, emotional and sensorial processes connected to the most significant belief (highlighted in bold), working accordingly and on the basis of the behavioral changes noticed during the elaboration phase. The integration with EMDR allowed access to significant past memories, in which the patient experienced a connection between incompetence—which was physiological at the time of the events—and fear, lack of control, and the impossibility of relying on a safe reference (the mother, more scared than her, asking her for help). The second target highlights the connection, in the patient’s experience, between spontaneous behavior and the other person’s unpredictable and angry reaction, an experience that conditioned beliefs related to the domains of control and safety. These events contributed to creating an excessive fear of uncertain situations in the patient, and an irrational fear regarding expectations and standards about how competent and autonomous a person must be to face situations. This prevented her from experimenting and living through unforeseen events and errors with relative tranquillity, relying on the possibility of repair and the idea that “learning comes from mistakes.” Also, in light of a specific case formulation related to the typical regulation characteristics of neurodivergence, and to favour an adequate modulation of emotional intensity, it was decided to alternate stimulation sessions with frontal ‘commentary’ sessions, allowing for the cooperative and shared monitoring of the load of stimuli to be processed. This sought to maintain a good balance between the patient well-being and the use and regulation of the synthesis and integration capacities necessary for the ongoing processing. Furthermore, to meet the needs related to her attentional and sensory characteristics, it was preferred to use tapping (administered by the clinician) alternated with the butterfly hug (self-administered by the patient with the clinician’s guidance), rather than bilateral eye movement stimulation, which proved burdensome in terms of attentional focus and sensory load. Physical distance and type of bilateral stimulation were always agreed upon with the patient’s needs, as well as with respect to clinical and strategic efficacy. The targets were identified and shared with the patient in light of the internal profile of functioning and in integration with the use of EMDR, also in relation to the impact that autism can have on factors connected to the perception and processing of traumatic experience (sensoriality, overload, overwhelming, metacognition). These adaptations were integrated with constant work on self-monitoring and regulation of the emotional and physiological state (tolerance window). During the processing, the work with EMDR led to a profound modification of the sensations and emotions related to the unprocessed relational trauma aspects, which had been powerfully re-stimulated by the triggers that the lived situations represented, especially in relation to the connected schemas of autonomy, competence, control, and safety. The integration of therapy with EMDR brought overall benefits to the patient’s perceived sense of efficacy and the demonstrated anxiety symptomatology. Regarding the phases of the EMDR protocol, specific strategies were selected, and variations were used to contextualize the application of EMDR always in relation to the work on autism, developing certain adjustments. The specific adaptations concern Phase 1 of the protocol, related to the use of salient life history episodes and their focus within the patient’s psychological functioning, and Phase 2, during which ample space was given to the psychoeducational and preparatory process. In the preparatory and psychoeducational phase, the adjustments were useful to maintain a ‘virtuous’ balance between information load and integration/processing processes, also through an extension of the time window dedicated to this phase. This allowed the patient to acquire a greater sense of mastery and knowledge of the path and protocol, familiarizing herself with the steps and the sensations experienced (awareness, in this sense, became a resource). Furthermore, the use of visual material (graphic sheets; maps; resources) was supported, which is useful for sustaining the patient’s processing capacity during moments of increased load (emotional, sensory, cognitive, and metacognitive). The use of strategies based on the polyvagal theory was introduced at this stage of the work, to promote their integration and the stabilization of the patient.

In the subsequent phases, although the standard protocol was used, specific adjustments were included, both in the assessment phase (Phase 3) and in the desensitization phase (Phase 4). During Phase 3, the targets were dynamically identified and placed within the patient’s internal profile of functioning, also in relation to the impact that autism can have on factors connected to the perception and processing of traumatic experience. Information regarding the relationship between neurodivergent characteristics, pathogenic schemas, and trauma processing, and the peculiarities of this dynamic in ASD, was also constantly shared, with references to the psychoeducational phase. A further adaptation involved alternating EMDR stimulation sessions with conversational sessions throughout the protocol (particularly during Phase 4, Desensitization). This was done to assess the need for integrative interventions and to allow for cooperative monitoring—along with the patient—of the emotional, cognitive, and sensory load experienced during the meetings. Moreover, as previously specified, to meet the needs related to her attentional and sensory characteristics, we opted to use tapping (administered by the clinician) in alternating integration with the Butterfly Hug (self-administered by the patient with clinician guidance), rather than bilateral eye movement stimulation, which proved taxing in terms of attentional focus and sensory load. Session by session, SUD values decreased, reaching 1 and VOC increased, reaching a perceived score of 7. Throughout all sessions, the patient demonstrated excellent compliance, and the adjustments were tailored to her needs. There were no adverse reactions, blockages, shutdowns, or meltdowns, and stabilizing practices during and between sessions—such as breathing or grounding exercises, self-paced and managed by the patient—helped manage increased emotional, cognitive, and sensory overload.

## 4 Discussion

The present clinical case aims to illustrate how certain modifications to the classic EMDR protocol can be useful in adapting it for autistic individuals. In particular, the use of strategies based on Polyvagal Theory, combined with adaptations of the EMDR protocol specifically for the sensory and metacognitive characteristics of autistic individuals, appears to be particularly effective clinically. The use of these adaptations is consistent with the theory of adaptive information processing (AIP), on which EMDR is based, and with the safety levels required to functionally restore the processing process. Although present in the literature, there is currently not much evidence regarding the use of EMDR in ASD. Leuning et al. observed some benefits from a 10-session EMDR treatment on communication and social awareness domains, though these were assessed by the subjects’ parents (Leuning et al., 2023). A recent Delphi Consensus also focused on the barriers that can make EMDR treatment less accessible and beneficial for autistic people. Among these, we can indeed include typical patient characteristics (such as communication difficulties and emotional regulation issues), but also characteristics related to therapists, such as a lack of confidence in using EMDR with this population (Fisher et al., 2023). Furthermore, some evidence currently focuses instead on the beneficial role of EMDR treatment for parents of autistic children, in order to alleviate related stress as well as any associated feelings of guilt (Rashidi et al., 2025). As previously noted, autistic people demonstrate heightened susceptibility to trauma. Moreover, the phenotypic presentation of the disorder may hinder the accurate clinical assessment of trauma-related conditions, resulting in subsequent misdiagnosis and the utilization of suboptimal rehabilitation strategies (Palmer and Dvir, 2024). Furthermore, despite the perception of trauma being similar between autistic individuals and neurotypical individuals, the former experience greater exposure to interpersonal trauma and a higher incidence of stress symptoms (Lim and Young, 2025). Adding to this is the increased propensity for autistic people to experience even minor events as traumatic, experiences that would not be classified as such according to the DSM-5 diagnostic criteria (Rumball et al., 2020). The vulnerability to trauma might also be rooted in anomalous sensory characteristics, which are one of the core symptoms of ASD: specifically, hyper-reactivity to external stimuli can lead to a cascade of elevated anxiety levels, which in turn may compromise an individual’s competence to counteract or cope with stressors (van Lobregt- Buuren et al., 2021). Differences in the detection of threat and safety signals, rooted in alterations in the autonomic nervous system found in autistic people, could also help explain the hyperreactivity to anxiety and stress and the associated greater psychopathological weakness, also in relation to the effect of trauma and intervention strategies. Some of the EMDR changes used in this case, such as flexibility, are similar to those already suggested by other studies and therapeutic research for autistic patients. The novelty of this case report lies in some specific aspects, which we summarize here. First, the order of the work steps: EMDR is explained and introduced only after awareness work on autism and in conjunction with knowledge of individual characteristics in terms of sensoriality and emotional regulation. Furthermore, in conjunction with awareness work, emotional and autonomic modulation exercises are used to promote stabilization, a sense of personal safety, and elements of overload, so as to remain within one’s window of tolerance. This was done based on the case formulation and in relation to specific neurodevelopmental differences, allowing for the use of polyvagal strategies as heuristics, combining biofeedback signals with the use of images of the window of tolerance to monitor one’s state and the therapeutic work in a shared manner. Then, all the targets were identified and shared with the patient in light of the internal profile of functioning and in integration with the use of EMDR, also in relation to the impact that autism can have on factors connected to the perception and processing of traumatic experience (sensoriality, overload, overwhelming, metacognition). These adaptations were integrated with constant work on self-monitoring and regulation of the emotional and physiological state (tolerance window). Also the decision to “tolerate” more negative cognitions is also a specific practice of our proposal, as is the possibility of “accepting” low SUD scores above “0″ if clinical reasoning suggests, as in this clinical report, they stem from residual overload (to be addressed with dedicated strategies) and not from incomplete reprocessing. So, our case report aims to introduce specific adaptations that make trauma-related experiences treatable in autism, where a lack of adaptation and the lack of preventive and specific intervention strategies addressing sensory differences and responses to stress and threat can make traumatic conditions difficult to treat in this population. Research on EMDR for autistic people is still very limited, with mostly small and uncontrolled studies. The case report highlights a clinical heuristic based on available treatment evidence and clinical reasoning, within a neurodivergence-informed perspective. Further studies are needed, with pre- and post-treatment parameters and larger clinical samples, to pave the way for the formulation of intervention protocols. The authors intend this to be the direction of their work.

## 5 Patient perspective

The patient is currently continuing therapeutic work, and the benefits derived from EMDR have remained stable over time. The bottom-up and associative EMDR approach profoundly modified the pathogenic beliefs and the emotional and sensory information linked to the traumatic experiences. It was this more radical and profound modification that generated a noticeable change in the patient’s behavior, with a positive impact on her capacity to cope with novel current situations. In these situations, the patient now demonstrates greater tranquility, more flexible coping strategies that are spontaneously oriented toward exploration, and are less influenced by feelings of vulnerability and distrust. Consequently, her avoidance and hyper-control behaviors, as well as inhibition, surrender, or flight reactions, are also reduced. The patient stated at the end of EMDR sessions: “Now I no longer feel alone and helpless. I realized that that intense fear was a memory; it was me remembering without knowing it. Now, every time a new situation scares me, it’s as if I can see that small, frightened part of me and I can attend to it. I don’t quite know how it happened, but something has fundamentally changed inside me. The best feeling is being more secure, capable of reacting. It’s a beautiful achievement for me.”

## Data Availability

The original contributions presented in the study are included in the article/supplementary material, further inquiries can be directed to the corresponding author.
